# Costs of switching auditory spatial attention in following conversational turn-taking

**DOI:** 10.3389/fnins.2015.00124

**Published:** 2015-04-20

**Authors:** Gaven Lin, Simon Carlile

**Affiliations:** Auditory Neuroscience Laboratory, Department of Physiology, School of Medical Sciences, University of SydneySydney, NSW, Australia

**Keywords:** spatial attention, speech, cocktail party, switch costs, working memory, cognitive load

## Abstract

Following a multi-talker conversation relies on the ability to rapidly and efficiently shift the focus of spatial attention from one talker to another. The current study investigated the listening costs associated with shifts in spatial attention during conversational turn-taking in 16 normally-hearing listeners using a novel sentence recall task. Three pairs of syntactically fixed but semantically unpredictable matrix sentences, recorded from a single male talker, were presented concurrently through an array of three loudspeakers (directly ahead and +/−30° azimuth). Subjects attended to one spatial location, cued by a tone, and followed the target conversation from one sentence to the next using the call-sign at the beginning of each sentence. Subjects were required to report the last three words of each sentence (speech recall task) or answer multiple choice questions related to the target material (speech comprehension task). The reading span test, attention network test, and trail making test were also administered to assess working memory, attentional control, and executive function. There was a 10.7 ± 1.3% decrease in word recall, a pronounced primacy effect, and a rise in masker confusion errors and word omissions when the target switched location between sentences. Switching costs were independent of the location, direction, and angular size of the spatial shift but did appear to be load dependent and only significant for complex questions requiring multiple cognitive operations. Reading span scores were positively correlated with total words recalled, and negatively correlated with switching costs and word omissions. Task switching speed (Trail-B time) was also significantly correlated with recall accuracy. Overall, this study highlights (i) the listening costs associated with shifts in spatial attention and (ii) the important role of working memory in maintaining goal relevant information and extracting meaning from dynamic multi-talker conversations.

## Introduction

In a cocktail party environment, listeners are faced with the challenging task of separating multiple simultaneous talkers overlapping in time, frequency, and space. The auditory system is able to parse this complex mixture into meaningful perceptual objects (Griffiths and Warren, 2004) using perceived differences in spatial location (e.g., Freyman et al., 1999; Kidd et al., 2005) as well as non-spatial cues such as voice characteristics and prosody (e.g., Darwin and Hukin, 2000; Brungart et al., 2001; Darwin, 2008). These features drive selective attention and allow listeners to focus on one talker of interest while filtering out competing talkers and noise (Shinn-Cunningham, 2008; Carlile, 2014 for reviews).

Cocktail party environments however are rarely static. In a multi-talker exchange, the focus of a conversation constantly shifts from one talker to another. Listeners must be able to rapidly reorient their selective attention in order to follow a conversation. Although non-spatial cues are important in initial auditory grouping, differences in *spatial* location drive temporal streaming particularly in complex multi-talker settings (Shinn-Cunningham, 2005; Allen et al., 2008; Ihlefeld and Shinn-Cunningham, 2008). Little is known about the perceptual consequences of switching *spatial* attention especially in dynamic conversations which involves integration of information across space and time (Sacks et al., 1974; Hutchby and Wooffitt, 2008).

Spatial attention operates like a searchlight, where processing resources can be allocated to a particular region or item in space. This spotlight of attention is limited and there is a gradient where attention falls off as a function of distance from the attended source (Mondor and Zatorre, 1995; Allen et al., 2009). There are benefits of knowing where and when to listen (Kidd et al., 2005; Kitterick et al., 2010) and any deviations from expectancy can lead to a reduction in speech intelligibility. This is consistent with Brungart and Simpson (2007), who showed that performance in a dynamic listening task decreased as a function of spatial transition probability. There has been strong evidence to suggest that auditory attention is object based and that representations build up over time (e.g., Best et al., 2008). Consequently shifts in stimulus location or a change in the attended-to voice result in a cost in streaming performance (Best et al., 2008, 2010).

These studies all highlight the benefit of spatial continuity in auditory object formation and establish that there is a cost associated with switching attention, even when switches are cued and predictable (Best et al., 2010; Koch et al., 2011). Reorientation of spatial attention is critical in the context of following conversations, yet little is known about the processes which drive this. Previous multi-talker studies have been limited to non-complex stimuli such as tones, digits, and simple speech corpora such as the co-ordinate response measure (CRM). However, this is not truly reflective of the cognitive demands of real world listening, which requires multiple element retention and semantic integration across space and time.

Over the past decade, increasing literature has been devoted to unraveling the role of cognition in cocktail party listening (Akeroyd, 2008; Arlinger et al., 2009 for reviews). In particular working memory, the capacity to hold and manipulate task relevant information (Baddeley, 2003; Engle and Kane, 2004), has been central to understanding how we interact with the world around us. Working memory is important for selective attention (de Fockert, 2013), hypothesis generation (Francis and Nusbaum, 2009) and suppressing the effect of distracters (Sörqvist, 2010; Hughes et al., 2013).

Studies in the visual domain (Kane and Engle, 2003; Caparos and Linnell, 2010; Ahmed and de Fockert, 2012) and the auditory domain (Conway et al., 2001; Dalton et al., 2009) have shown that working memory and processing load affect the spatial window of attention. Maintaining task relevant information is dependent on the precision of this selective attention, which influences the degree of distracter processing (Lavie et al., 2004; Lavie, 2005; de Fockert, 2013). As working memory demands increase, performance begins to decline in selective attention tasks, which results in a rise in subjective listening effort (Rönnberg et al., 2014). The recently, proposed ease of language understanding (Rönnberg et al., 2008, 2013) and cognitive spare capacity (Rudner et al., 2011; Mishra et al., 2014) models posit that listeners have a finite pool of working memory resources, which can be allocated to encoding, rehearsal, and comprehension of stimuli. The greater the cognitive load, the less residual resources available for processing of information. Ultimately, complex auditory scenes not only present a challenge in terms of selective attention but also cognitive demands, which influence the fidelity of recall.

This study aimed to investigate the cost of switching spatial attention during conversational turn-taking. We aimed to explore the relationship between attention switching and cognitive processes including working memory in normally-hearing listeners. Word recall and discourse comprehension were examined using matrix sentences (Hagerman, 1982) in a novel paradigm involving speech rehearsal and spatial reorientation. Matrix sentences, which are syntactically fixed but semantically unpredictable, have low stimulus redundancy and allow for the examination of recall independent of context. These structured sentences are particularly appealing for this study as they better approximate the content, semantic diversity and working memory demands of a real world conversation compared to digit recall or predictable closed set sentences found in CRM speech.

Experiment 1 investigated six word recall following a single endogenous switch in spatial attention. Matrix sentences from three concurrent sources were used to isolate spatial switching costs. All three sources were drawn from recordings of the same talker to control for non-spatial cues such as voice characteristics, thereby forcing listeners to rely on spatial information to separate and drive selective attention. Performance in trials involving a switch in target location between two sentences was compared to trials with a non-shifting target. The target location was varied to investigate whether recall differed as a function of the size, spatial hemisphere (left vs. right), and direction of the shift. It was hypothesized that there would be a decrease in recall following a shift in target location, due to a disruption in auditory streaming (Best et al., 2008, 2010) and attentional reorientation following target search (Kidd et al., 2005; Brungart and Simpson, 2007). In addition, cognitive functions including working memory capacity were hypothesized to be correlated with total words recalled and distractor processing during conversational turn-taking.

Experiment 2 was designed as a follow-up to Experiment 1, and investigated the effect of increasing processing load on sentence comprehension. Comprehension of speech relies not only on effective recall but a combination of processes including; segregation of competing streams, discrimination of words, and semantic processing at the sentence level. These processes are important in adverse listening conditions, particularly when listening in demanding situations with high levels of informational masking. This experiment aimed to investigate whether switching performance was load dependent, consistent with a working memory hypothesis. Rather than assessing simple word recall, this experiment used performance on questions related to the content of the sentences to assess the extent of semantic processing. If working memory is involved in attention switching then we would anticipate an increase in switching cost with increasing question difficulty.

## Materials and methods

### Participants

Sixteen young normally-hearing listeners (9 male, aged 21–35, *M* = 23.9, *SD* = 4.0) participated in two auditory attention switching experiments. All listeners had English as their first language, normal hearing as assessed by a pure-tone audiogram (<20 dB hearing loss at frequencies between 250 and 8000 Hz), and no reported cognitive or attentional deficits. All subjects gave written informed consent in accordance with the Human Research Ethics Committee, University of Sydney.

### Setup

Experiments were conducted in a sound attenuated audiometric booth (2.5 × 2.4 × 2.2 m in dimension). Listeners sat with their head fixed on a chin rest facing an array of three Tannoy Active loudspeakers, positioned at eye level 1 m from the head at –30, 0, and 30° azimuth.

### Stimuli

**Figure 1:**
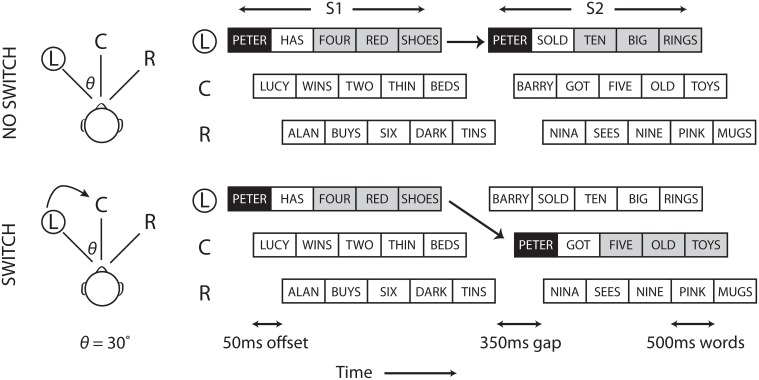
**Experimental setup**. Matrix sentences were presented over loudspeakers positioned to the left (L), center (C), and right (R) of the listener's head. Examples are shown for a single no switch (top), and switch (bottom) trial. Subjects attended to the cued location (circled) and followed sentences (S1 and S2) with the cued name, in this case “Peter”. In Experiment 1, subjects were required to verbally recall the last three words of each target sentence (gray). In Experiment 2, subjects answered a graded multiple choice question related to the target sentences.

Three pairs of matrix sentences, recorded from a single male Australian English talker, were presented from the three loudspeaker locations (Figure 1). Matrix sentences were syntactically fixed and comprised of name, verb, number, adjective, and noun elements. Sentences were constructed at each trial by randomly sampling each element without replacement from a list of 10 possible words. All words within a trial occurred only once, with the exception of the target name which occurred twice.

Words were 500 ms in duration with the exception of nouns, which were time stretched to 600 ms using Adobe Audition 3.0. This manipulation was applied to reproduce the natural prosodic lengthening of speech at phrase boundaries (Wightman et al., 1992). A 350 ms silence gap was introduced between sentence pairs to replicate the average conversational turn-taking duration of English speech (Stivers et al., 2009). In addition, sentences were staggered with a 50 ms offset to (i) reduce the effects of energetic masking encountered with synchronized concurrent talkers and (ii) enhance grouping by staggering onsets. Offset combinations were randomized each trial and balanced for all locations. Stimuli were generated using Matlab (MathWorks) and played through an RME FireFace UCX soundcard at 48 kHz sampling rate. All sentences were presented at 65 dB SPL.

### Procedure

Both experiments utilized the same setup and stimuli but differed in their post stimulus task. Each trial began with a 0.75 s 500 Hz priming tone presented from one of three loudspeakers. Subjects directed their spatial attention to this cue and were instructed to remember the name and sentence that followed at this location. A second set of matrix sentences were presented after a silent turn-taking gap. Subjects were required to search for and attend to the sentence with the same target name, which either remained in the same spatial location (no switch trials) or moved to another spatial location (switch trials). There were three possible target locations for the first sentence (S1) and three possible target locations for the second sentence (S2), yielding a total of nine possible spatial conditions. The target sentence was presented with equal likelihood at all loudspeaker locations. Subjects performed one of two tasks at the end of each trial depending on the experiment.

#### Experiment 1: speech recall

Subjects were required to verbally report the last three words of each target sentence in correct serial order (six item recall). Subjects also reported the target name to verify that they followed the correct stream. Only trials where subjects correctly identified the name were included in analysis (83.5% of trials). Verbal responses were recorded using a microphone and saved for scoring and analysis after the experiment. If a subject could not recall a word during a trial, this was registered as a “pass”. Subjects completed a short training block to familiarize themselves with the stimuli and procedure before starting a total of 24 repeats for nine spatial conditions in randomized order (4 blocks of 54 trials).

#### Experiment 2: speech comprehension

Subjects were presented with two multiple choice questions on a computer screen following each trial (one for each target sentence). Questions varied in complexity ranging from 1-Step simple recognition questions e.g., which word was in the target sentence? to 2-Step specific recall questions e.g., which big item did Peter sell? to 3-Step quantity comparison questions e.g., which item had the smallest/largest number?. These questions were based on those used by Rönnberg et al. (2014). Subjects were required to respond as fast and as accurately as possible using a keypad. Subjects participated in a short training block before completing total of 6 repeats for 9 spatial conditions and 3 question types in randomized order (3 blocks of 54 trials).

### Cognitive tasks

Subjects also completed a battery of cognitive tests including the reading span test to measure working memory capacity (Daneman and Carpenter, 1980; Baddeley et al., 1985), attention network test to measure attentional modulation (Fan et al., 2002), and trail making test to measure executive function (Reitan, 1958).

In the reading span test, subjects were presented with a series of short sentences on a computer screen, starting with 3 and increasing in length to 6. Participants were required to read the sentences out aloud and verbally report whether each made literal sense or not (half were non-sensical). At the end of each series, subjects were prompted to recall either the first or last words of each of the sentences. The number of total correct words recalled was used as a measure of working memory capacity.

The attention network test was a cued reaction time flanker task presented on a computer screen. Subjects attended to a fixation cross at the center of the screen which was accompanied by an arrow above or below the fixation point. Subjects were required to respond as fast and as accurately with the left or right keyboard keys to indicate the direction of the arrow. A number of conditions were tested including with congruent/incongruent flanking arrows, with/without a temporal alerting cue, and with/without a target spatial cue. Three measures of attentional control were extracted from the test; alerting ability, orienting ability, and cue conflict resolution.

The trail making test consisted of two timed pen and paper tests which required subjects to connect a series of labeled circles in ascending numerical order (Trail-A) or alternating numeric-alphabetical order (Trail-B). These two tests provide coarse measures of visuo-motor processing and task switching speed, respectively, while the difference score (Trail-B minus Trail-A) provides an estimate of executive control ability (Sánchez-Cubillo et al., 2009).

### Data analysis

#### Center correction

A score correction was applied to all conditions containing a central target, to account for the energetic disadvantage posed by the absence of an acoustic “better-ear” (Zurek, 1993). This disadvantage was estimated for each subject as the difference between the central no switch condition (CC) and the mean of the left (LL) and right (RR) no switch conditions. The full correction was applied to the CC condition, while half of this correction was applied to conditions which contained one central target (LC, CL, CR, and RC).

#### Error analysis

In addition to measuring the number of words correct, the errors committed by each subject were analyzed for their relative frequency. In Experiment 1, “masker confusions” and “passes” were calculated for each condition to quantify the degree of informational masking and failures in word recall, respectively. Masker confusions were instances where a subject reported a word presented in a concurrent masking stream, while passes were instances where a subject failed to register a response for a particular word.

In Experiment 2, subjects were presented with multiple choice questions with one correct option and two incorrect options. For 1-step questions, incorrect options included a masker confusion and an unspoken word (a word which was not presented in the trial and was reflective of random guessing). For 2- and 3-step questions, incorrect options included a masker confusion and a sentence order confusion (a word which was present in the target stream but was embedded in the alternate sentence). The latter type of error occurred when subjects mixed words from sentence 1 and 2, reflecting a failure to integrate information.

#### Statistical analysis

Data from Experiment 1 were normally distributed. The mean number of words correct for each spatial condition were compared using a repeated measures One-Way ANOVA. No switch trials were compared with corresponding switch trials using a series of planned paired *t*-tests. Switching costs were calculated for each subject as the mean difference in performance between no switch and switch conditions. Further analysis was performed on recall rates, masker confusions, and passes using Three-Way repeated measures ANOVAs examining the effects of word, sentence position, and switching. The relationship between listening task performance and cognitive test scores were examined using linear correlations.

Data from Experiment 2 were not normally distributed and were arc-sine transformed. This transformation converts binomial data into an approximately normal distribution for parametric analysis (Studebaker, 1985). Performance was analyzed using Two-Way repeated measures ANOVAs with task difficulty and switching as independent variables. The difference between switching and no switching performance were analyzed for each question type using paired *t*-tests. Outliers were not removed from either experiment.

## Results

### Experiment 1

#### Total words recalled

**Figure 2:**
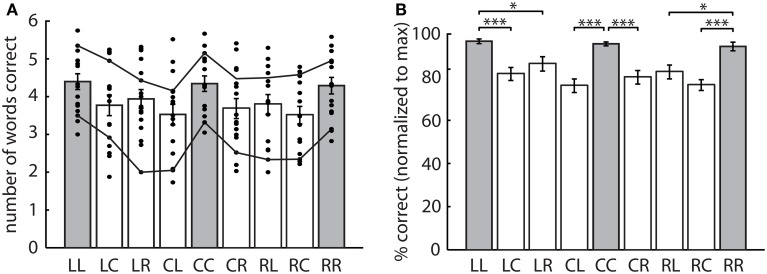
**Experiment 1 scores. (A)** Mean number of words correct for nine spatial conditions. Large variability in individuals scores (dots) was observed but trends across conditions were similar between high and low performing subjects (solid lines). **(B)** Normalized percentage correct for nine spatial conditions. Performance was significantly higher for no switch (gray) compared to switch trials (white). Bars represent mean ± SEM, ^*^p < 0.05, ^***^p < 0.001.

There was considerable variability in performance between individuals in the speech recall task (Figure 2A). Scores ranged from 1.7 to 5.8 words correct per trial (out of 6), with differences as large as twofold between subjects in certain conditions. Despite this variability, trends across conditions were similar, with mean performance higher in no switch trials compared to switch trials.

Scores were consistently higher for some subjects than for others. To better examine the within-subjects effect of switching, the number of words correct was normalized to the maximum score for each subject (Figure 2B). A One-Way repeated measures ANOVA on normalized data confirmed a significant effect of spatial condition [*F*_(4.5, 67.9)_ = 12.5, *p* < 0.001]. Planned pairwise comparisons indicated a significant recall advantage in no switch trials compared to respective switch trials (LL > LC, LR; CC > CL, CR; RR > RL, RC). There were no significant differences between any of the switch conditions, demonstrating no effect of location, direction, and angular size of the spatial shift on word recall. Overall, switching spatial attention resulted in a 10.7 ± 1.3% decrease in word recall when averaged across subjects and locations.

#### Sentence and word recall

**Figure 3:**
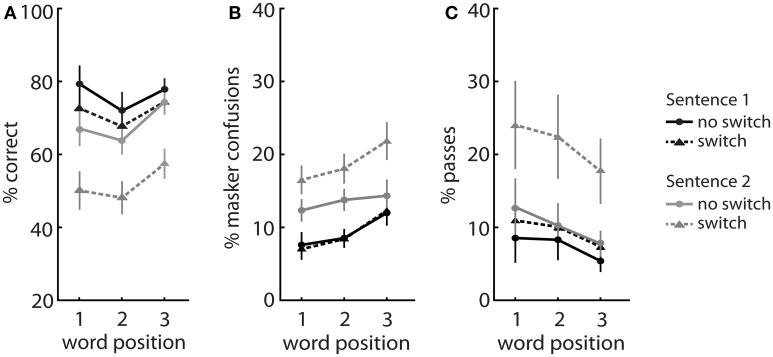
**Experiment 1—The effect of sentence and word position on (A) words correct, (B) masker confusions, and (C) frequency of passes, during no switch and switch trials**. Data presented as mean ± SEM.

A Three-Way repeated measures ANOVA on percent correct data revealed a significant main effect of sentence number [*F*_(1, 15)_ = 20.0, *p* < 0.001], word position [*F*_(2, 30)_ = 6.3, *p* < 0.01], and switching [*F*_(1, 15)_ = 69.0, *p* < 0.001]. Recall was lower for the second target word and for the second target sentence (S2) in each trial, particularly following a switch in spatial attention (Figure 3A). There was a significant sentence by switch interaction effect [*F*_(1, 15)_ = 10.8, *p* < 0.01], where recall dropped significantly between S1 (71.6 ± 4.5%) and S2 (51.9 ± 4.6%) in the switch condition (*p* < 0.001). In contrast, there was minimal decline in recall between S1 and S2 in the no switch condition (76.4 ± 4.0 vs. 68.4 ± 3.4%, respectively, *p* > 0.05).

The effect of word position resembled a classic serial position curve (Figure 3A), with recall greatest for the first and last items in each sentence. A significant sentence by word interaction effect [*F*_(1, 30)_ = 3.8, *p* < 0.05] was observed, where the final target word was recalled significantly more often than the second target word (68.6 ± 2.3 vs. 60.5 ± 3.8%, *p* < 0.01) for S2 only. This word recency effect was less pronounced in S1 but was observed in both switch and no switch conditions.

#### Masker confusions

A Three-Way repeated measures ANOVA on masker confusions revealed a significant main effect of sentence number [*F*_(1, 15)_ = 36.9, *p* < 0.001], word position [*F*_(2, 30)_ = 21.0, *p* < 0.001], and switching [*F*_(1, 15)_ = 12.3, *p* < 0.01], and a significant sentence by switch interaction effect [*F*_(1, 15)_ = 17.9, *p* < 0.01]. Masker confusions constituted ~9–19% of responses and were most prevalent in the final word of each sentence, and primarily in S2 (Figure 3B). There was no significant difference in the frequency of masker confusions between sentences in the no switch condition. However, the number of masker confusions doubled from S1 (9.2 ± 1.2%) to S2 (18.8 ± 1.8%) following a switch in spatial attention (*p* < 0.001).

Interestingly, listeners demonstrated significantly greater masker confusions for the last word (15.1 ± 1.6%), compared to the first (10.9 ± 1.2%, *p* < 0.001) and the second target words (12.2 ± 1.1%, *p* < 0.01) in each sentence. We speculate that this may be a “masker” recency effect.

#### Passes

A Three-Way repeated measures ANOVA on pass rates revealed a significant main effect of sentence number [*F*_(1, 15)_ = 10.6, *p* < 0.01], word position [*F*_(1.3, 18.9)_ = 4.2, *p* < 0.05], and switching [*F*_(1, 15)_ = 14.1, *p* < 0.01], and a significant sentence by switch interaction effect [*F*_(1, 15)_ = 7.2, *p* < 0.05]. Passes were more prevalent for the first word of each sentence, and for S2 overall (Figure 3C). The frequency of passing remained below 12% in the no switch condition, and there was no significant difference in pass rates between the first and second sentences (7.4 ± 2.4 vs. 10.3 ± 2.4%, *p* > 0.05). However, the likelihood of passing increased twofold for S2 when there was a switch (21.4 ± 5.2%), compared to S1 pre-switch (9.4 ± 3.0%, *p* < 0.05).

Passes in the second sentence were not always due to a failure in search. Subjects were able to recall at least one correct word from S2 in 87.7% of no switch trials and 69.5% of switch trials. This implies that they were able to locate the second sentence in the majority of trials. A supplementary experiment was devised using the same paradigm but without recall of elements, to test the ability to simply follow the target with minimal cognitive load. In this experiment, a subset of six subjects was able to locate S2 with a high success rate, 93.1% of the time during no switch trials and 88.4% of the time during switch trials.

#### Cognitive correlates

Correlations between Experiment 1 performance and cognitive test scores for the cohort are shown in Table 1. The number of words correct per trial were positively correlated with reading span score (*r* = 0.46, *p* < 0.05) and negatively correlated with Trail-B time (*r* = −0.46, *p* < 0.05). Reading span score was also negatively correlated with switching costs (*r* = −0.44, *p* < 0.05) and frequency of passes (*r* = −0.57, *p* < 0.05). There were no significant correlations between any measure of the attention network test and performance in the listening task. Other measures of the trail making test were also not correlated with listening performance.

**Table 1:** **Pearson correlation coefficients between Experiment 1 scores and cognitive test scores**.

	**RST score**	**ANT-A**	**ANT-O**	**ANT-C**	**Trail-A**	**Trail-B**	**Trail B-A**
Words correct	0.457^*^	0.057	0.219	−0.320	−0.390	-0.458^*^	−0.307
Switching cost	-0.442^*^	−0.316	−0.252	0.176	0.389	0.396	0.245
Masker confusions	0.423	0.054	0.109	−0.043	−0.019	0.348	0.361
Passes	-0.565^*^	−0.261	−0.290	0.417	0.413	0.166	0.002

*RST, Reading Span Test; ANT, Attention Network Test Alerting (ANT-A); Orienting, (ANT-O); Conflict resolving ability, (ANT-C); Trail, Trail making test A (Trail-A), test B (Trail-B), and difference score (Trail B-A)*.

* *p < 0.05 shown in bold*.

### Experiment 2

#### Percent correct

**Figure 4:**
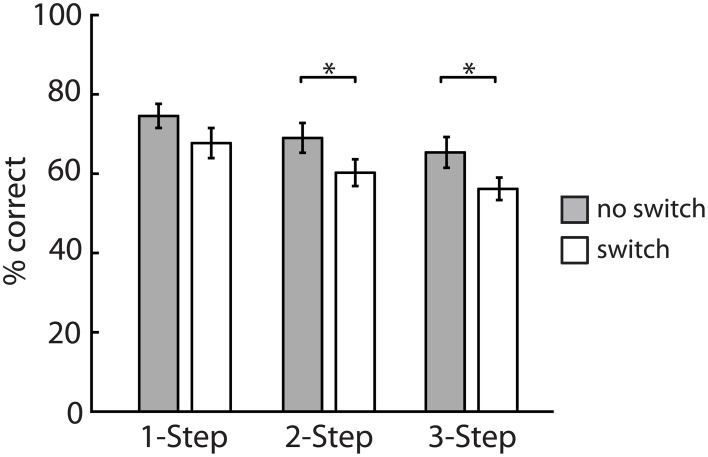
**Experiment 2—Percentage of correct responses for three question types**. Switching costs were significant only for 2 and 3-step questions. Bars represent mean ± SEM, ^*^p < 0.05.

Experiment 2 was designed as a follow-up to Experiment 1, to explore the effect of increasing processing load on switching costs. A Two-Way repeated measure ANOVA on percent correct data revealed a significant main effect of switching [*F*_(1, 15)_ = 21.2, *p* < 0.001], and a main effect of question type [*F*_(2, 30)_ = 12.7, *p* < 0.001] on correct responses, but no significant question by switch interaction. Sentence comprehension decreased for switch trials and decreased with increasing question complexity (Figure 4). Switching costs were load dependent and increased proportionally with the number of cognitive operations in each question (6.9, 8.5, and 9.2% cost for 1-step, 2-step, and 3-step questions, respectively). Planned pairwise comparisons revealed a significant switching cost only in the 2-step [*t*_(15)_ = 3.4, *p* < 0.05] and 3-step conditions [*t*_(15)_ = 2.2, *p* < 0.05] but not in the 1-step condition [*t*_(15)_ = 2.3, *p* > 0.05].

#### Sentence analysis

**Figure 5:**
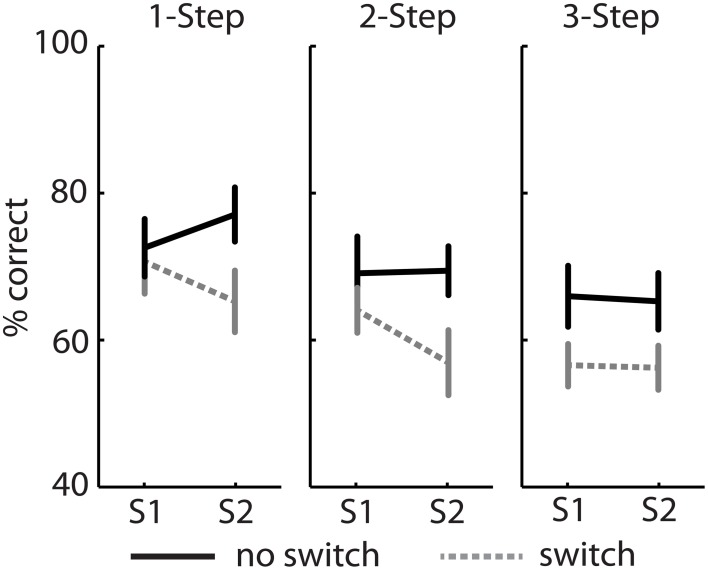
**Experiment 2—Percentage of correct responses from sentence 1 (S1) and 2 (S2) for three question types**. Bars represent mean ± SEM.

A Three-Way repeated measures ANOVA revealed that there was no significant main effect of sentence on performance [*F*_(1, 15)_ = 1.1, *p* = 0.3]. The sentence by switch interaction was non-significant [*F*_(1, 15)_ = 3.1, *p* = 0.098]. As seen in Figure 5, performance was higher for S1 compared to S2 only under certain conditions. Trends were similar to those observed in Experiment 1 with a small sentence primacy effect evident following a switch in both 1-step and 2-step conditions. This effect was however abolished following a complex 3-step question.

#### Error analysis

**Figure 6:**
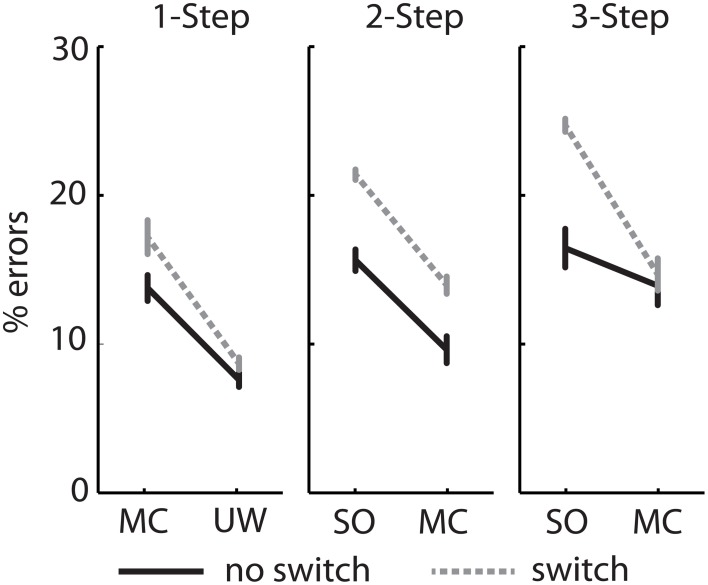
**Experiment 2—Proportion of masker confusion (MC), unspoken word (UW), and sentence order (SO) errors for three question types**. Data presented as mean ± SEM.

There were greater errors committed in the switch condition compared to the no switch condition (Figure 6). For simple 1-step questions, subjects were more likely to report masker confusions than unspoken words (with a guess rate of <10%). For complex 2- and 3-step questions, sentence order confusions were more prevalent than masker confusions. Switching spatial attention increased the proportion of all error types in the 1- and 2-step conditions. However, in the 3-step condition, switching resulted in a disproportionate increase in sentence order confusions but not of masker confusions. Thus, as question load increased, subjects tended to make less location attribution errors (confusing competing streams) and more semantic attribution errors (confusing elements from S1 to S2).

## Discussion

This study examined the cost of switching endogenous spatial attention in a dynamic three talker cocktail party setting. In a cohort of young normally-hearing listeners, there was a significant decrease in word recall and discourse comprehension following a switch in target location in a two sentence selective attention paradigm. The cost was independent of the location, direction, and angular size of the spatial shift and was predominantly confined to the second sentence post switch. The drop in recall was associated with a concomitant increase in reported masker confusions and word omissions. The significant relationship between listening task performance and reading span score supports the hypothesis that switching efficacy is driven by working memory. An individual's working memory capacity impacts their ability to accurately recall words across space and time. This study also demonstrates that there is a cognitive load associated with switching attention during conversational listening. Systematic increases in question difficulty lead to a progressive decline in switch performance, providing evidence that attention switching is both load and working memory dependent.

### The cost of switching spatial attention

Switching spatial attention resulted in a decrease in word recall. The costs observed in this study are within range of previous reported switching costs of 5–15% by Best et al. (2008) using five talkers, and up to 15% observed by Brungart and Simpson (2007) using three talkers. One key difference in this study is the use of a single male talker at all three locations, to control for the influence of non-spatial cues including voice characteristics. Although not ecological, this manipulation isolates the cost of a single endogenous switch in spatial attention using relatively diverse conversational stimuli.

Previous studies have attributed switching costs to target location uncertainty (Kidd et al., 2005; Brungart and Simpson, 2007) and disruption to object streaming continuity (Best et al., 2008, 2010). The reduction in recall, predominantly confined to the second sentence, post switch, supports this notion. This drop in performance however, cannot be solely attributed to a failure to re-engage or find the second sentence as subjects could report at least one correct word from this sentence 78.6% of the time. This implies that cost in this paradigm was not primarily due to location uncertainty, but perhaps other factors such as disruption to streaming or cognitive load. Indeed in a supplementary experiment, without any cognitive load, subjects were able to localize the second target sentence with 90.7% accuracy.

We propose two possible mechanisms for this degradation in second sentence recall. Firstly, cognitive load from word rehearsal may decrease efficiency of the switch and subsequent search for S2. Based on the difference between no load and load identification of S2 (88.4 vs. 69.5%), there does appear to be some evidence for a degradation in localization as a result of rehearsal. Consequently, subjects were more likely to commit masker confusions or pass in S2 as they were unable to identify the target stream. Alternatively, the reduction in recall fidelity may be due to increased cognitive load induced by the switch itself. In Experiment 2, we see that switch costs are not uniform and are load dependent. Systematic increases in post presentation question difficulty amplified the cost of switching, supporting a limited working memory model. Furthermore, analysis of the errors revealed the prevalence of sentence order confusions over masker confusions implying successful stream segregation but unsuccessful attribution of semantic details. Thus, subjects were able to localize the correct target during the switch but were unable to integrate information in the post-stimulus decision phase. This is strong support for the notion of switching increasing cognitive load.

Neither the distance, direction, nor location of the spatial shift had any significant bearing on performance. These results are consistent with the findings of Mondor and Zatorre (1995) and Brungart and Simpson (2007), who demonstrated that performance in a spatial orienting task did not decline as a function of shift distance and angular displacement size. It appears that the average turn taking gap of 350 ms (Stivers et al., 2009) was sufficient to allow subjects to reorient their attention up to 60° in this paradigm. This duration is well outside the timeframe of 80–200 ms proposed by other studies for spatial reorientation (Teder-Salejarvi and Hillyard, 1998). Under the no-load condition, subjects were able to redirect their attention with high success rate during the conversational gap. It was only under load that this performance decreased. Time is a critical factor for speech understanding (Singh et al., 2008, 2013; Koch et al., 2011; Dhamani et al., 2013), particularly in multi-talker conversations which involve rapid and unpredictable shifts in target location.

In Experiment 1, the lack of an interaction between word position score and switching condition demonstrates that there was no temporal impact of the switch on word recognition immediately post switch. Koch et al. (2011) showed that there was a delay associated with having to switch attention between ears in a dichotic listening task. However, there was no significant “inertia” observed in our performance data. The uniform drop in recall across all three words suggests that elements were equally susceptible to interference rather than a failure to reorient attention fast enough.

Even though we did not observe any location dependent costs, it should be noted that scores observed in this study were adjusted with a center correction. The center correction is an estimate of the energetic disadvantage posed by the absence of a better ear. This correction may however *overestimate* the performance disadvantage posed by a central talker flanked by two maskers, and thus *underestimate* the true switching cost when presented with a central target. In addition, the performance disadvantage may not be additive in all switching conditions.

### Individual differences

Notably, we found large individual differences in task performance in this cohort of young normally-hearing listeners. Correlations between switching performance and individual cognitive measures strongly support the theory that working memory is important for maintaining task relevant information in adverse listening conditions (Baddeley, 2003; Engle and Kane, 2004). The positive correlation between number of words correct and working memory capacity reinforces the importance of information retention and manipulation for comprehension during dynamic conversations. Furthermore, the negative correlation between switching costs and working memory highlights the disparity between high and low working memory individuals in their ability to retain information across switches. High working memory subjects are not only better at selective attention tasks (Conway et al., 2001) but have been shown to be more proficient at divided attention tasks which involve monitoring the occurrence of a target name across multiple streams (Colflesh and Conway, 2007).

However, contrary to previous predictions, working memory was not associated with distractor processing as suggested by some studies (Conway et al., 2001; Ahmed and de Fockert, 2012). Switching attention did increase the overall proportion of masker confusions (Figure 3B), but this was not associated with individual cognitive correlates. This may be due to the type of distraction encountered in this task. Recent studies propose a duplex theory of distraction which posit that an irrelevant stream can either (i) capture attention due to stimulus deviation or (ii) interfere with serial rehearsal due to the changing state of the distractor stream (Hughes, 2014; Sörqvist and Rönnberg, 2014). The former, but not the latter, has been shown to be correlated with working memory capacity (Sörqvist et al., 2013). It is quite possible that non-target streams interfered with the process of rehearsal rather than attention capture in this experiment. Another potential explanation may lie in the nature of the task, which permitted the absence of responses (“passes”). Reporting masker confusions was thus dependent on the discretion and response criterion of the subject. Interestingly, analysis of pass frequency was associated with the second sentence switching performance (Figure 3C) and negatively correlated with an individual's working memory (Table 1). This suggests that decay of rehearsed information found in this study may be related to information storage capacity.

The other significant correlation was between total words recalled and Trail-B time, which is a measure of task switching ability (Sánchez-Cubillo et al., 2009). Perhaps not surprisingly, faster task switching meant better performance in our listening task- which inherently involves a switch from selective to divided, back to selective attention. Visuo-motor processing (Trail-A) and executive function (Trail B–A) were perhaps not as prominent in this listening task, however some correlations were bordering significance.

The lack of a correlation between any of the measures of the attention network test may have two explanations. While the ANT may be effective in revealing differences in clinical populations such as in ADHD (Johnson et al., 2008), the test has less resolution in this cohort of young healthy subjects. Secondly, the test examines basic attentional modulation and not attentional capacity under load, the latter of which is most important when dealing with multi-talker cocktail party environments. Neither of the three measures of the ANT were driving the effects we were observing in our listening test, which were primarily working memory and task switching based. It should also be noted that the tests employed in this study are not mutually exclusive and there may be some overlap between cognitive processes.

Furthermore, differences in performance may depend on the type of strategy adopted by the individual listener. Studies have shown that the probability of target locations has an influence on the allocation of attention and consequently speech intelligibility (Kidd et al., 2005; Brungart and Simpson, 2007). The current task, where all locations are equally probable as targets, requires both selective and divided attention, and is reflective of an unpredictable, uncued conversation. Interestingly, subjects were found to distribute their expectations evenly during the conversational gap. Following a switch in target location, almost half (48.9%) of reported masker confusions in S2 arose from the original S1 target location while the other 51.1% originated from the non-target location. This provides evidence that the no switch advantage was not due to subjects simply keeping their attention fixated on the S1 location.

### The importance of working memory

Working memory involves the storage, manipulation and recall of goal-relevant information, and the inhibition of distracters. This study reinforces the notion of conversational tracking as an active task which requires cognitive resources, especially when there is a shift in spatial attention. This supports both the cognitive spare capacity model proposed by Rudner et al. (2011) and ease of language understanding model by Rönnberg et al. (2008, 2013).

Based on these models, working memory is limited and must be allocated to various components of the listening task. Here working memory is important in encoding, rehearsing, and recalling information across switches in spatial attention. Individuals with low working memory capacity can only encode a limited amount of information and have little residual “spare capacity” to process the information, hence lower recall. The introduction of a switch requires allocation of cognitive resources and further limits spare capacity to encode and recall information particularly in S2. Individuals with high working memory capacity experience these constraints to a lesser extent. Furthermore, studies have shown that subjects with better cognitive abilities including working memory, distracter inhibition, and text reception threshold have better speech intelligibility, selective attention, and word recall in noise (Kjellberg et al., 2008; Koelewijn et al., 2012; Meister et al., 2013).

In Experiment 2, increases in cognitive load had implications for broader discourse comprehension. Based on the ease of language understanding model, higher working memory load leads to a decrease in the fidelity of encoded information which impacts lexical access and downstream comprehension (Rönnberg et al., 2008, 2013). This has implications not only for normally-hearing listeners but for elderly and hearing impaired listeners with peripheral and cognitive deficits. Working memory deteriorates with age and there is greater cognitive load and effort following hearing loss (Tun et al., 2009). Peripheral deficits lead to a myriad of downstream deficits including elevated thresholds, failure to group and segregate sounds, poorer speech intelligibility, and greater central processing demands.

In real world listening we rely on semantic information and contextual cues to endogenously guide attention (Pichora-Fuller et al., 1995; Meister et al., 2013). The use of a fixed syntax, unpredictable corpus allows for examination of sentence comprehension while removing the influence of context. While this is advantageous in a controlled environment for isolating recall costs, in real world situations context plays an important role in stream formation and discourse comprehension (Pichora-Fuller et al., 1995). Context is believed to alleviate some of this cognitive load associated with listening in adverse conditions as it allows for top-down prediction of words (Rönnberg et al., 2013).

Another potential contributing factor not measured in this experiment is the level of proactive interference experienced by each subject. Proactive interference refers to the degradation of memory traces by prior encoded information (Kane and Engle, 2000), particularly items with a similar context—such as, words within the same category in a closed set corpus. The ability to resist semantic proactive interference has been shown to be closely related to speech in noise recognition (Ellis and Rönnberg, 2014). Differences in this study in the level of proactive interference between high and low working memory participants may mediate cross-trial or within-trial interference and hence the accuracy of recall. The increase in masker confusions and sentence order confusions following a switch may reflect an increase in interference from previously encoded sentences. However, these errors are difficult to quantify in the current study as the same words can be present in multiple successive trials.

## Conclusion

Switching spatial attention in a cocktail party setting imposes a cognitive load which impacts short term recall of words. This cognitive load impacts the disengagement and reorientation of attention and consequently the encoding of information immediately following the switch. This has a downstream effect on comprehension of sentences in a multi-talker conversation. Switching led to an increase in distractor interference and higher likelihood to miss words. Costs appear to be direction, spatial hemisphere, and size independent but do seem to be load dependent and only significant with tasks involving multiple operations. These results support the notion of a limited working memory model which is involved in directing spatial attention, encoding, and post-perceptual processing of stimuli in a multi-talker auditory scene.

### Conflict of interest statement

The authors declare that the research was conducted in the absence of any commercial or financial relationships that could be construed as a potential conflict of interest.
